# Dietary Grape Pomace Supplementation in Dairy Cows: Effect on Nutritional Quality of Milk and Its Derived Dairy Products

**DOI:** 10.3390/foods9020168

**Published:** 2020-02-10

**Authors:** Andrea Ianni, Giuseppe Martino

**Affiliations:** Faculty of Bioscience and Technology for Food, Agriculture and Environment, University of Teramo, Via R. Balzarini 1, 64100 Teramo, Italy; aianni@unite.it

**Keywords:** grape pomace, dairy cow, milk, cheese, antioxidant, polyunsaturated fatty acid, linoleic acid, volatile compound

## Abstract

Grape pomace (GP) is the main solid by-product of winemaking and represents a rich source of potent bioactive compounds which could display a wide range of beneficial effects in human health for their association with reduced risk of several chronic diseases. Several studies have proposed the use of GP as a macro-ingredient to obtain economically worthwhile animal feedstuffs naturally enriched by polyphenols and dietary fibers. Moreover, the research carried out in this field in the last two decades evidences the ability of GP to induce beneficial effects in cow milk and its derived dairy products. First of all, a general increase in concentration of polyunsaturated fatty acids (PUFA) was observed, and this could be considered the reflection of the high content of these compounds in the by-product. Furthermore, an improvement in the oxidative stability of dairy products was observed, presumably as a direct consequence of the high content of bioactive compounds in GP that are credited with high and well-characterized antioxidant functions. Last but not least, particularly in ripened cheeses, volatile compounds (VOCs) were identified, arising both from lipolytic and proteolytic processes and commonly associated with pleasant aromatic notes. In conclusion, the GP introduction in the diet of lactating cows made it possible to obtain dairy products characterized by improved nutritional properties and high health functionality. Furthermore, the presumable improvement of organoleptic properties seems to be effective in contributing to an increase in the consumer acceptability of the novel products. This review aims to evaluate the effect of the dietary GP supplementation on the quality of milk and dairy products deriving from lactating dairy cows.

## 1. Introduction

Grape (*Vitis* spp.) represents one of the most consumed and appreciated fruits in the world. As previously reported by Zhu et al. [1], approximately 75% of the entire production is utilized for wine-making, which accounts an annual worldwide production of just under 30 billion liters, mainly from *Vitis vinifera*, with Italy, France and Spain that, in that order, representing the main producers [2].

As in all agro-industrial sectors, a significant production is generally associated with the relevant accumulation of difficult-to-degrade by-products, management of which represents an issue of great importance due to its environmental impact [3]. The need for ecologically sustainable and economically advantageous disposal of agro-industrial by-products has led over time to the development of specific research that has enhanced these matrices, highlighting their high biotechnological potential and paving the way for alternative uses, especially as food supplements for humans and mainly for farm animals [4].

With particular regard to this last aspect, several experimental feeding strategies have been developed over time and administered mainly to ruminants, but also to pigs, chickens and laying hens [5]. Overall, the use of matrices of plant origin, particularly rich in bioactive compounds, has led to interesting effects both on animal welfare and on quantitative and qualitative aspects of animal productions. For instance, the use of olive pomace, the main by-product of olive oil production, as feed supplement for dairy cows results in effective improvement of the health functionality of milk and its derived dairy products [6], and has been observed to induce positive effects on inflammation and cholesterol in laying hens [7].

Grape pomace (GP) is the solid by-product of wine-making, approximately representing 20% of the total processed grapes, and has been reported to be a rich source of biologically active compounds, especially polyphenols, to which is attributed the ability to interfere with several biological mechanisms, determining positive effects for human health [8]. Specifically, the dietary intake of these compounds has been demonstrated to reduce the risk of the onset of chronic pathological conditions, such as inflammatory and neoplastic diseases [9,10]. In addition to polyphenols, particular interest has also been given to the recovery of antioxidant fibers from skin [11,12], as well as oil extraction from seeds [13]. Over time, all these matrices have undergone a thorough characterization with a view to evaluating their possible use as macro-ingredients for the production of novel functional food products enriched with bioactive compounds [14,15,16].

In the last two decades, the use of grape pomace as a dietary supplement for farm animals has been the focus of numerous experimentations. The RNA sequencing-based whole-transcriptome profiling of Friesian calves that have received a dietary supplementation with dried GP showed a reduced expression of genes coding for cholesterol biosynthesis enzymes. This finding has also been consolidated by the reduction of blood cholesterol levels and an improvement of oxidative stability in carcasses [17]. In ewes the ability of dietary GP to induce an immune-modulatory function has been reported, without adverse effects on milk production [18]. In lambs, the GP intake significantly increased the activity of reduced glutathione and catalase in blood and tissues, contributing to the reduction of oxidative damage in lipids and proteins. In addition to this, in fecal microbiota, an enhanced growth of facultative probiotic bacteria and the concomitant inhibition of pathogen populations such as *E. coli* was observed [19]. GP was also demonstrated to increase the diversity of the rumen microbiota in dairy calves [20], although in sheep the GP dietary intake was reported to impoverish the cellulolytic and proteolytic bacteria population in rumen, with consequent reduction of microbial protein yield [21]. To highlight the wide range of experimentation carried out by feeding farm animals with GP, an interesting finding was also represented by the role of this agro-industrial by-product in improving the membrane integrity and overall quality of boar semen during storage, presumably as a consequence of reduced lipid peroxidation of ejaculated spermatozoa induced by GP polyphenols [22].

In addition to what has been reported, the effect of dietary GP on qualitative and quantitative parameters of animal product has also been the subject of numerous studies. Specifically, much attention has been paid to the evaluation of the chemical-nutritional characteristics of milk and its derived products. Specifically, the objective of this review is to briefly recall the main properties of GP, and to then focus attention on its use as a dietary supplement in ruminants, giving particular emphasis to the effect that these feeding strategies have on the quality of dairy products, both fresh and ripened.

## 2. Grape Pomace Constituents: Chemical Composition and Biological Properties

GP mainly consists of grape seeds and skins. Seeds are rich in compounds credited of antioxidant activity, such as phenolic acids, flavonoids and procyanidins, while grape skins contain abundant anthocyanins. GP is also characterized by non-negligible amounts of lipids, proteins, minerals and fiber. Specifically, grape seeds have an oil content ranging from 15% and 18%, which is rich in essential fatty acids, non-digestible carbohydrates, proteins and non-phenolic bioactive compounds such as tocopherols and β-carotene [8]. These characteristics make this by-product particularly interesting due to the potential health benefits for humans, and for this reason it has attracted over time great interest from the nutraceutical sector.

### 2.1. Polyphenols in GP

#### 2.1.1. Chemical Composition

**Figure 1:**
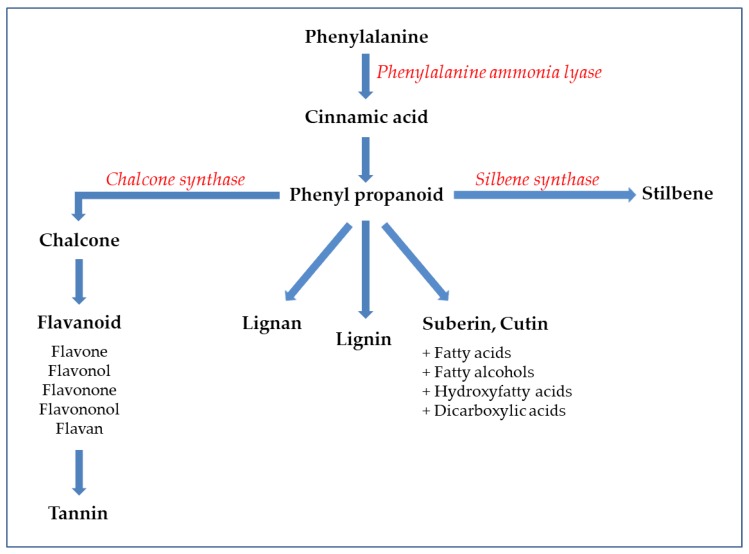
Schematic representation of flavonoids, tannins, stilbenes, lignans, lignins, suberins and cutins from phenylalanine.

Phenolic compounds are secondary metabolites of plants, characterized from a chemical point of view by an aromatic residue wherein one or more hydroxyl substituents are present. These compounds are classified into different classes taking into account the similarity of their chemical structures and their precursor is mainly represented by phenylalanine (Figure 1), and only in a few cases by tyrosine. The polyphenols of greatest interest in the food sector are largely divided into four classes, consisting of phenolic acids, flavonoids, lignans and stilbenes [23].

**Figure 2:**
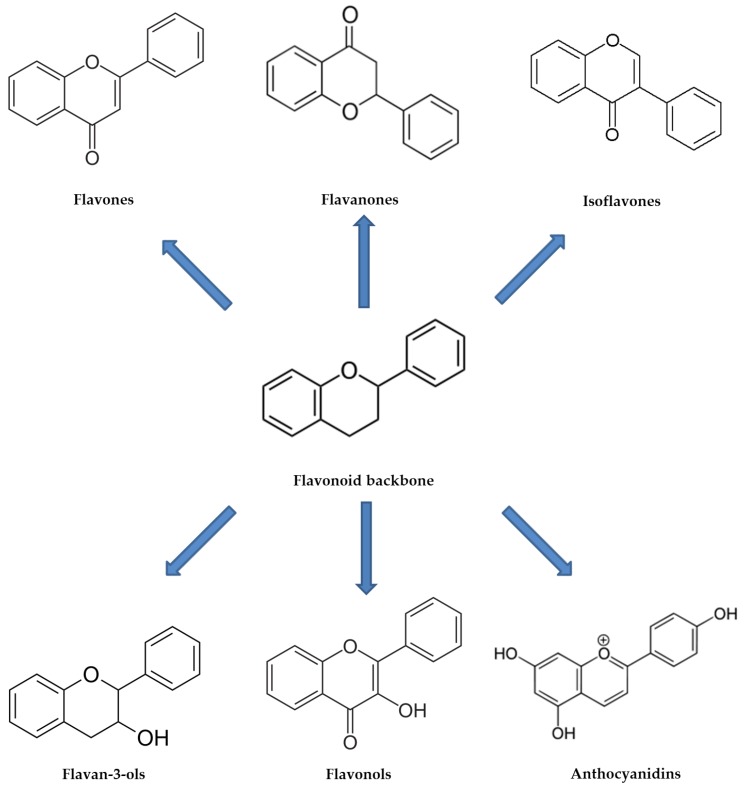
Representation of basic structures of flavonoid subclasses.

Phenolic acids are characterized by a carboxylic functional group and are commonly found in the form of hydroxycinnamic acids. This category consists of several compounds, including gallic, ferulic, caffeic and coumaric acids, which are commonly found as glycosylated derivatives [24]. Flavonoids certainly represent the most studied and best characterized polyphenols, and among them are listed flavones, isoflavones, flavonols, flavanones, anthocyanidins/anthocyanins, flavanols and condensed tannins (Figure 2), distinguished on the basis of the chemical structure [25].

Polyphenols are present in virtually all matrices of plant origin, but some fruits, more than others, are particularly rich in these bioactive compounds, as in the case of grape and apple. As reported by Makris et al. [26], GP is characterized by high concentrations of extractable polyphenols (approximatively 10% on a dry matter basis), specifically phenolic acids, anthocyanins, catechins, procyanidins, flavonols, and stilbenes [27] whose exact composition is, however, strongly dependent on grape variety. Generally, the red varieties are characterized by high concentrations of anthocyanins, while in white varieties the flavan-3-ols (gallocatechin, procyanidin B1, procyanidin B2, procyanidin B4, procyanidin C1, catechin and epigallocatechin) have been reported to be the most abundant polyphenols [28,29]. Polyphenol composition also tends to vary in different parts of the grape. In grape skins, mainly hydroxycinnamic acids, flavanols, flavonol glycosides and anthocyanins are represented, which are greatly influenced by vinification method and contact time. Grape seeds, on the other hand, are reported to be essentially rich in gallic acid and flavan-3-ols, which easily condense into oligomeric and polymeric compounds, as in the case of condensed tannins [30].

**Figure 3:**
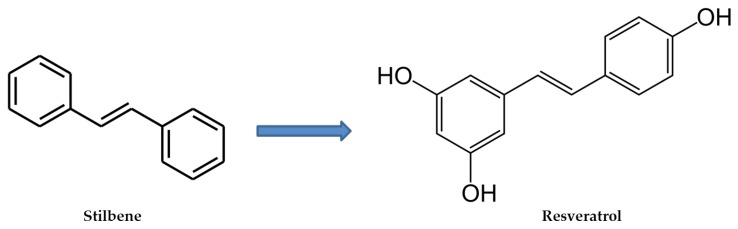
Structures of stilbene and resveratrol.

Another relevant bioactive compound found both in skins and seeds is represented by resveratrol (Figure 3), the specific content of which in grapes is influenced not only by the grape variety, but also by plant maturation [31]. Resveratrol and its glycosides represent the major stilbenoids, and are phytoalexins synthesized in plants as a consequence of a pathogen attack. Its importance for humans is related to its low toxicity and well-characterized anti-inflammatory and fungicidal activity [32]. An important finding concerns the fact that during wine-making, higher resveratrol concentration tends to remain in GP, only being transferred to wine in small percentages [33].

Evaluations performed on the indigestible fraction of GP showed the presence of high concentrations of condensed tannins (about 16.0% in white grape skins and 27% in red grape skins), and non-starch polysaccharides, mainly cellulose and pectins, ranging from 17% to 21% on a dry matter basis. In addition to this, a relevant percentage of protein (up to 80%) was also shown to be indigestible in vitro [34].

#### 2.1.2. Biological Properties

In recent decades, a large research area has been focused on the study of the biological properties associated with polyphenolic compounds. With specific regard to the food sector, this phenomenon has developed in response to the growing interest shown by consumers towards products accredited with nutraceutical properties, and therefore functional in the prevention of various pathological conditions, if regularly taken (Table 1).

Numerous classes of polyphenols isolated from GP have exhibited interesting biological properties in both in vitro and in vivo studies. Procyanidins, particularly represented in grape seeds, have been demonstrated to promote important antioxidant, anti-inflammatory and anti-carcinogenic activities. Kulisic-Bilusic et al. [35] provided evidence of the antioxidant activity of these compounds in HT-29 human colon cancer cells, while Packer et al. [36] reported in their review their ability to act as free radical scavengers, counteracting reactive oxygen and nitrogen species; furthermore, several cardiovascular benefits have been described as a consequence of vasorelaxant activity [37], inhibition of the activity of angiotensin-converting enzyme, and the ability to improve the capillary permeability, enhancing microcirculation. In addition to this, Bak et al. [38] reported an in vitro study conducted on lipopolysaccharide-stimulated RAW 264.7 murine cells, in which procyanidins showed the ability to exert a potent anti-inflammatory activity through the regulation of the NFκB and p38 MAPK pathways, resulting in a decreased expression of inflammatory mediators, such as an inducible nitric oxide synthase and cyclooxygenase-2. Further studies on cell models characterized by neoplastic potential, also made it possible to highlight the cytotoxicity of these compounds towards human breast, lung, gastric adenocarcinoma cells, at the same time improving growth and viability of gastric mucosal cells [39].

It has been reported that phenolic compounds obtained both from grape skin and seeds are also able to modulate the function of several matrix metalloproteinases (MMPs), zinc-dependent enzymes with endopeptidase activity which are involved in a wide range of physiological and pathological events associated with the turnover of the extracellular matrix [40]. La et al. [41] showed a grape seed extract to reduce the secretion and extracellular activity of MMP-1 and MMP-9 in lipopolysaccharide-stimulated macrophages, presumably as a consequence of the inactivation of NF-κB p65 and AP-1 pathway. Tyagi et al. [42] also reported decreased secretion of MMP-2 and MMP-9 after treating human prostate carcinoma DU145 cells with a grape seed extract. The anti-carcinogenic effect was explained by advancing the hypothesis of a direct role of the treatment in inhibiting the phosphorylation of proteins belonging to the MAPK family and consequently the NFκB activation.

Grape seed proanthocyanidins have also been reported to promote apoptosis in in vitro studies conducted on non-small cell lung cancer A549 and H1299 cells. This finding has been explained by assuming an increase in the expression of Bax, a proapoptotic factor, and a decrease in expression of antiapoptotic mediators belonging to the Bcl family, with the consequent alteration of mitochondrial membrane potential and activation of caspases 3 and 9 [43].

One important bioactive compound, particularly represented in GP and to which have been attributed numerous functions from a biochemical point of view, is certainly resveratrol. Overall, voluminous evidence has been collected regarding the ability of this compound to prevent or slow down the onset and progression of several pathological events, such as neoplastic conditions, cardiovascular diseases, ischemic injuries, chronic inflammations, and infections [44]. In light of all this, and as also reported in the review of Shukla and Singh [45], all the collected preclinical findings have been helpful in characterizing the chemopreventive function of resveratrol, which can therefore be considered to be an effective tool in countering cancer. In addition to this, resveratrol, in the same way as quercetin and catechin, has been reported to reduce plasma cholesterol in hamsters, in which was also observed the development of several mechanisms effective in preventing atherosclerosis [46].

Taking into account the outcome of numerous epidemiological studies, the consumption of foods particularly rich in phenolic compounds showed effective in breaking down the risk of the onset of cardiovascular diseases, commonly associated with the alteration of fatty acid metabolism and the increase of the lipid oxidative damage. Low concentrations of plasma antioxidants are generally responsible for the increase of low-density lipoprotein (LDL) oxidation, whose products are implicated in the biochemical mechanisms responsible for artery blockage and thrombosis. Polyphenols obtained from grape seeds have been shown to be able to lower the risk of heart disease, specifically by inhibiting the LDL oxidation [47]. The inhibition of LDL oxidation also represents one of the mechanisms by which grape phenolic compounds can mitigate atherosclerosis, presumably as a consequence of the inhibition of platelet aggregation, the reduction of inflammation and the expression of proteins credited to slow down cell senescence [48]. Furthermore, research has been conducted over the years on grape flavonoids as key elements in the development of nutraceuticals. In this regard, the review of Georgiev et al. is very informative [49], discussing the scientific advances arising from the research in the phytochemical field, leading to the identification of grape flavonoids as ideal candidates for the production of nutraceuticals because of their marked and well-characterized antioxidant, anti-inflammatory and antiproliferative properties. With specific regard to quercetin, several studies have reported this compound to be effective in suppressing intracellular ROS formation, MMP expression and activation, and cell motility in in vitro studies [50,51].

Other phenolic compounds credited with numerous biological functions, and highly represented in grapes, are catechins (Table 1). Such compounds and their derivatives have been reported to act in vitro as scavengers of reactive oxygen species, in addition to the well-characterized antioxidant function, which is indirectly influenced through the regulation of transcription factors and enzyme activities. In humans, only a modest and transient increase in plasma antioxidant potential has been evidenced as a consequence of dietary catechins intake; however, promising results have been obtained from studies on animal models in which the effects on biomarkers of oxidative stress were evaluated, with specific regard to oxidative DNA damage [52]. To what has just been reported for catechins, can be added antibacterial [53], anti-inflammatory [54] and antineoplastic effects [55].

Tannins represent a large group of compounds conventionally classified into hydrolysable and condensed. For a long time, tannins were considered to negatively influence the animal physiology. In any case, their specific effect strongly depends on various factors, including the type of consumed tannin, its chemical structure and molecular weight, the ingested amount, and the animal species involved. Feeding strategies characterized by high concentrations of this compounds have been reported to reduce the voluntary feed intake and nutrient digestibility, whereas moderate tannin intake may improve feed utilization as a consequence of a decrease in ruminal protein degradation and subsequent increase in concentration of amino acids in the small intestine.

These variations in nutrition have been clearly demonstrated to induce effects on animal performances. Sczechowiak et al. [56] showed the ability of a diet rich in condensed tannins to modify microbial population in rumen in lactating cows. This obviously had effects on rumen fermentation and biohydrogenation, consequently inducing significant variations in the fatty acids profile of milk. The authors specifically reported an increase in the concentration of C18:1 *trans*11 (vaccenic acid) as a consequence of the inhibition of the last steps of rumen biohydrogenation, thus preventing reduction to C18:0. Furthermore, in a similar study was also reported an increase in concentration of vaccenic acid and *n*-3 fatty acids in plasma, thus generating a favorable condition for the accumulation of polyunsaturated fatty acids (PUFAs) in the mammary gland and consequently in milk [57].

With specific regard to the GP effect on ruminal microbiota, the study of Biscarini et al. should be mentioned [20], in which dairy calves received a diet enriched with 10% DM (dry matter) of red GP for 75 days. The metagenomic approach on rumen liquor evidenced a taxonomic enrichment mainly associated with *Ruminiclostridium* and *Eubacterium* sp., whose functions were related to degradation of GP constituents, such as flavonoids and xyloglucan. Interestingly, the authors also reported variations in the lipopolysaccharide biosynthetic pathway, supposedly as a result of antimicrobial effects.

Despite the well-characterized antioxidant properties, phenolic compounds are credited also with a pro-oxidant potential, which tends to occur in the presence of certain environmental conditions. Castañeda-Arriaga et al. [58] discussed the key aspects involved in determining the balance between antioxidant and pro-oxidant effects, and particular attention has been paid to pH, presence of redox metals and the possibility of phenolic compounds to be converted into benzoquinones.

### 2.2. Fatty Acid Composition and Antioxidant Properties of Grape Seed Oil

As previously mentioned, grape seeds are characterized by an oil content approximately ranging between 15% and 19% depending on grape variety and maturity. The specific fatty acids composition of grape seed oil is also strongly influenced by variety and maturity. In a study conducted by Lutterodt et al. [59], Chardonnay, Muscadine, Ruby red, and Concord grape seed oils were analyzed for fatty acid composition. Linoleic acid (C18:2) was reported to be the major fatty acid (66.0–75.3%), followed by oleic (C18:1; 13.9–21.9%), palmitic (C16:0; 7.05–7.75%) and stearic (C18:0; 2.52–4.72%) acids. This finding is in agreement with most of the related literature. Beveridge et al. [60] reported a C18:2 concentration ranging from 66.8% to 73.6% in seven distinct seed oils obtained from as many grape varieties. The authors also reported a C18:1 concentration ranging from 12% to 19%, with lower values for C16:0 and C18:0. Similarly, Ianni et al. [61] evidenced a C18:2 concentration equal to 71.59% by analyzing GP obtained from an Italian grape variety. Other authors, however, reported variable values for the mentioned fatty acids, presumably as a direct consequence of grape origin and method applied for the oil extraction. For instance, El-Shami et al. [62] reported higher values for C18:1 in Egyptian grape seeds, whereas Crews et al. [63] evidenced variable fatty acid profiles in seed oils obtained from Italian, French and Spanish varieties.

This matrix is therefore particularly rich in fatty acids that have been reported to induce several benefits for human health. Extensive literature highlighted that diets rich in of monounsaturated fatty acids (MUFA) is effective in promoting a healthy blood lipid profile, in modulating blood pressure, and positively controlling insulin sensitivity and glycemic index. In the same way, detrimental effects of dietary intake of saturated fatty acids (SFA) have been widely characterized [64]. With specific regard to linoleic acid, it has been shown in lactating ruminants that feeding strategies rich in this compound are effective in inducing an increase in concentration of conjugates of linoleic acid (CLA) in milk and derived dairy products. CLA are endogenously produced in ruminants starting from *trans*-11 18:1 (vaccenic acid) through an enzymatic mechanism mediated by Δ^9^-desaturase and, in addition to this, CLA also represent intermediates of the ruminal biohydrogenation of C18:2 taken by diet [65]. These compounds can be found almost exclusively in milk and dairy products and their beneficial properties for consumers health have led over time to develop experimental feeding strategies for ruminants in order to increase their concentration in animal productions [66].

In addition to the previously described phenolic antioxidants, grape seed oil is also characterized by non-phenolic antioxidants such as tocopherols and β-carotene, both vitamins of extreme interest for human health. In grape seed oil, α-tocopherol is generally the most abundant detected tocopherol, γ and δ-tocopherols were found in low concentrations, while β-tocopherol was not detected [67].

### 2.3. Fiber Content

The term “dietary fiber” (DF) was introduced to describe remnants of plant origin, resistant to hydrolysis by digestive enzymes [68]. DF is characterized by soluble and insoluble components, and the induced physiological effects strongly depend on the relative amount of these fractions. These compounds are mainly derived from the plant cell wall and among them, cellulose, hemicellulose and lignin should be mentioned [69]. In humans, soluble fraction has been reported to induce a decrease in plasma cholesterol and stem inflammatory diseases in gut; furthermore, a prebiotic function was observed to be able to preserve host health [70].

With regard to lactating ruminants, DF has a strong influence on ruminal activity, with direct effects on the animals’ welfare and milk quality, since the chemical composition of milk has been reported to be strongly influenced by the biochemical mechanism mediated by rumen microbiota. Both soluble carbohydrates and pectins undergo rapid degradation in the rumen, and only a small percentage of these compounds will be available for post-ruminal digestive mechanisms [71].

Non-extractable proanthocyanidins represent a not negligible partition of GP fiber. In a study conducted on rats, these compounds, following the achievement of the intestinal environment have been reported to be further hydrolyzed into smaller metabolites by the intestinal microbiota. This event was responsible for the release of phenolic acids that have been detected in urine as both free phenolic compounds and conjugates with glucuronate or sulphate residues. For that reason, authors hypothesized non-extractable proanthocyanidins to serve as a carrier for the progressive release of polyphenols which can be therefore absorbed in the distal portions of the intestine with an estimated bioavailability not less than 24 h after ingestion [72].

## 3. Role of GP Constituents in Food Systems

### 3.1. Food and Beverages Fortification

Over the past two decades several studies have been conducted with a view to exploiting the grape pomace constituents in the food sector. As previously reported, these compounds, especially polyphenols, are credited of numerous and interesting biological properties, with potential positive effects on consumer health [73]. In this regard, significant progress has been made in the direct inclusion of GP constituents in food, with the aim of exploiting GP as a polyphenol carrier able to induce an increase in concentration of phenolic compounds and an improvement of antioxidant potential, with undoubted advantages not only for consumers’ health but even for aspects related to the preservation of the food quality during storage.

GP constituents have been widely tested as fortifying agents in numerous and varied food preparations. Walker et al. [74] used GP from Pinot Noir and Pinot Grigio as a source of antioxidant dietary fiber to fortify baked goods, including breads, muffins, and brownies. The GP used to substitute the wheat flour at different concentrations was effective in inducing in the finished products an increase of total phenolic content, radical scavenging activity, and total dietary fiber, without any significant variations in sensory evaluations. In a similar study, Hoye and Ross [75] used grape seed flour in bread production. Against an increase in the total phenolic content, authors experienced a worsening of consumer acceptability after the addition of the higher concentrations of grape seed flour; specifically, a replacement of 100 g hard red spring wheat flour with 10 g of grape seed flour induced a decreased acceptance in relation to bitterness, astringency and sweetness. In the study of Peng et al. [76], bread was fortified with different amounts of grape seed extract (from 300 to 1000 mg per 500 g of bread) and antioxidant activity, texture and color of products were evaluated. Additionally, in this case an improvement of the antioxidant capacity in the experimental bread was reported, presumably as a consequence of the increase of total phenols; however, no sensorial evaluation was performed.

Several studies have also been conducted on the characterization of chemical and nutritional quality of fresh and ripened dairy products fortified with GP powders. Marchiani et al. [77] added GP powders from three grape varieties to semi-hard (Italian Toma-like) and hard cheeses (Cheddar). The authors evidenced no variations in proteolysis, microbial counts and physicochemical parameters; however, an increase of total phenolic content and radical scavenging activity was observed in ripened cheeses. Karaaslan et al. [78] prepared grape ethanol extracts which were used as functional ingredients for yogurt production. The obtained products showed high phenolic-anthocyanin content and exhibited an increased antioxidant power in comparison with control samples. Similarly, Tseng and Zhao [16] performed evaluation on yogurt supplemented with GP stored for 3 weeks at 4 °C. With respect to the control samples, the experimental yogurt showed an increase in pH and a decrease of viscosity, without variations in lactose concentration. In addition to this, the GP supplementation was also effective in reducing the peroxide values during storage, with advantages in oxidative stability. The production of functional yogurts was also pursued by Chouchouli et al. [79] who experimented the addition of grape seed extracts from two grape varieties (Moschofilero and Agiorgitiko). The fortification in the range of 5–10 mg of gallic acid equivalents for 100 g of yogurt did not affect pH and the count of *Lactobacilli*; furthermore, no significant variations were observed in the consistency, color and flavor compared to the control samples.

With specific regard to meat, the supplementation of 0.5–5% of grape seed flour in frankfurters was effective in improving the oxidative stability of experimental samples. Interestingly, an increase in concentration of protein, total dietary fiber and an improvement of water holding capacity was also highlighted [80].

The addition of phenolic extract also represents a practice of interest for the fortification of beverages. Aguilar et al. [81] proposed a study in which grape pomace, grape leaves and stems were used as functional matrices in order to enrich the must with phenolic compounds. This work was motivated by the consideration that, despite the relevant phenolic contents of grape, a major part of these compounds is lost in by-products during the different stages of wine-making. Grape juice enriched with extracts from pomace, leaves, and stems made it possible to obtain an antioxidant capacity that was considered at least as efficient as other phenols fortified beverages. Furthermore, all the selected by-products were effective in inducing an increase in concentration of polyphenols into the must, resulting in a beverage with promising antioxidant activity and potential health benefits for consumers.

Finally, the studies performed with the aim of characterizing grape pomace pigments with a view to their possible use as food colorants should also be mentioned. Especially in red grape, anthocyanins represent the phenolic compounds mainly responsible for the characteristic color of mature grapes. During winemaking, anthocyanins are partly extracted from the grape skins, determining wine pigmentation. Many of these compounds thus remain in the pomace, which may therefore represent a matrix of great interest for the recovery of pigments of considerable interest for technological food processes, in addition to the well-known nutraceutical properties [82,83].

### 3.2. Safety Issues Associated with GP Polyphenos Consumption

GP extracts are “Generally Recognized As Safe (GRAS)” matrices which can be used as colorants and antioxidant additives in flavored beverages [84]. However, as for all compounds with the ability to influence biological mechanisms, the pharmacological effects strictly depend on dose and are affected by several factors including age of consumers and genotype.

Mennen et al. [85] reported in their review that companies which produce and distribute nutritional supplements rich in phenolic compounds, recommend the consumption of 50 mg/day of isoflavones or 100–300 mg/day of grape seed extracts rich in proanthocyanidins. In addition to this, authors have also reported a series of aspects regarding the fact that consumption of relatively higher concentrations of these bioactive compounds can influence different biochemical processes, leading to undesirable effects. For instance, some phenolic compounds have been reported to induce carcinogenic and genotoxic effects, or may negatively influence the biosynthesis of the thyroid hormone. Consumption of polyphenols has also been observed to inhibit the recruitment of non-heme iron, with consequent depletion of the microelement in populations at risk. Furthermore, polyphenols may in some cases enhance the effect of several pharmacological agents, by interfering with their mechanism of action.

With specific regard to the zootechnical sector, the dietary supplementation of grape seed extracts in broiler chickens has been reported to induce a reduction of the intestinal length and an increase of the ileal digestibility of crude protein at 21 days of age. At 42 days of age, an increase of spleen weight was instead observed, without significant variations in animal performance and in the relative weights of liver and pancreas [86]. In the case of ruminants, it is necessary to refer to tannins, whose effects on animals can range from beneficial to toxicity and death. Tannins are commonly divided into two groups, hydrolysable and condensed, and are reported to induce both positive and detrimental effects in livestock, as a direct consequence of their concentration in feed, in addition to other parameters such as animal species, physiological state and composition of the whole diet. The observed negative effects of a feeding strategy particularly rich in tannins do not necessarily reflect the toxic potential of these compounds, but depend to toxicity resulting from remaining metabolized products, which cannot be further degraded by the animals’ detoxification mechanisms [87,88].

## 4. Grape Pomace as Feed Supplement in Dairy Cows: Main Properties of Derived Milk and Cheese

### 4.1. Chemical Composition of Milk and Cheese

The dietary intake of GP in lactating dairy cows has been shown to be able to modify the chemical properties of milk and its derived dairy products.

Chedea et al. [89] evaluated the effect of a diet supplemented with 15% GP on the health status and milk composition of dairy cows. The experimental diet did not induce significant variations in the total amount of milk fat and protein, important parameters for dairy products, but was effective in increasing the lactose concentration, a disaccharide synthesized by the mammary gland cells and commonly endowed with the ability to bind calcium, increasing its absorption. Regarding the analysis of milk protein fractions, higher concentrations of β-lactoglobulin were evidenced, but no effect was observed for α-lactalbumin, albumin and caseins. The β-lactoglobulin accounts for approximatively 50% of total whey proteins in cow milk, while it cannot be traced in human milk [90]. This protein is involved in several biological mechanisms, for instance its proteolytic digestion by trypsin has been reported to give origin to four peptides with low molecular weight credited of bactericidal activity; furthermore, hypocholesterolemic, antiviral and anticarcinogenic effects have been characterized [91]. The concomitant increase in concentration of lactose and β-lactoglobulin therefore assumes considerable importance, above all for the fact that these compounds represent the main constituents of the whey-derived powders, which have undergone widespread diffusion worldwide due to the interesting food functional attributes. With respect to this, the strong heat-set gelation properties of β-lactoglobulin should be mentioned, which is therefore widely used in the preparation of food products in which water-binding and texturization are crucial. In addition to this, the onset of specific interactions between β-lactoglobulin and lactose during whey powders preparation was demonstrated to be effective in avoiding the lactose crystallization that generally leads to detrimental events, mainly related to lipid oxidation [92].

In a study conducted by Ianni et al. [93], in which ten Friesian cows received for a dietary supplementation of 10% GP (on a dry matter (DM) basis) 56 days, the milk collected at the end of the trial did not show variations in chemical composition. This finding was also confirmed in pasteurized milk cheeses that were analyzed after 3, 7, 15 and 30 days from the cheese-making. In that case, slight modifications were observed only for proteolysis and were associated with the action of proteinases and peptidases released by the cheese microbiota. During the cheese manufacturing, milk was pasteurized, with the only microbial forms being represented by *Lactococcus* spp., *Lactobacillus* spp. and *S. thermophilus*, which were used as starters. Such micro-organisms are reported to be responsible for extensive proteolytic activity in cheese, with the consequent production of short peptides and free amino acids. The authors discussed this finding by assuming a role of GP bioactive compounds in favoring the metabolic pathways in lactic acid bacteria, leading to an increased function of proteolytic enzymes. In this regard is relevant what was previously reported by Viveros et al. [94], who showed the ability of a grape seed extract in increasing the growth of *Lactobacillus* at the expense of potentially harmful microbial forms, such as *Enterobacteriaceae* and *Clostridium*.

In the study of Ianni et al. [93] another interesting variation was represented by the increase in concentration of γ-aminobutyric acid (GABA) in cheese at the end of the ripening period. The increased GABA concentration was associated with a role of GP constituents in promoting the selection of specific species/strains, such as lactic acid bacteria, able to express several glutamate decarboxylases, responsible for catalyzing the decarboxylation of l-glutamate to GABA. In this regard, it is useful to recall studies in which GP and grape seed extracts were shown to be effective in inducing the growth of *Lactobacillus acidophilus*; furthermore, an environment rich in catechin and gallic acid was demonstrated to enhance the development of *Lactobacillus hilgardii* [95,96]. The results concerning the increase in concentration of GABA in dairy products, acquires particular relevance in consideration of the numerous potential benefits for consumer health, mainly related to blood pressure lowering, protection against chronic diseases, and immunity improvement under stress conditions [97,98].

### 4.2. Fatty Acid Profile

It is widely known that by modifying the feeding strategy, it is possible to change, to a certain extent, the relative fatty acids composition in milk and cheeses. This has also been shown to occur through the administration to lactating ruminants of grape by-products which, as previously reported, are particularly rich in linoleic acid. In a study conducted on ewes and goats, Tsiplakou and Zervas [99] showed that the administration of diets enriched with linoleic acid led to a significant increase in milk of fatty acids credited of greatest health benefits for humans, specifically vaccenic acid, linoleic acid, and conjugated linoleic acid.

The supplementation of lactating dairy cows’ diet with 10% of dried GP resulted in the effective induction of an increase in concentration of vaccenic acid (C18:1 *trans*-11) and linoleic acid (C18:2 *cis*-9, *cis*-12) in milk. The fatty acid profile of the derived cheese showed the same variations as evidenced in milk, with the addition of a significant increase in concentration of rumenic acid, a conjugated linoleic acid (C18:2 *cis*-9, *trans*-11) [100]. These findings are in agreement with those obtained from similar experiments in which grape by-products have been introduced in the diet of lactating ruminants. Correddu et al. [101] evidenced in ewes milk an increase in concentration of *n*-6 PUFA, especially linoleic acid, as a consequence of the addition of grape seed flour in the diet. Manso et al. [102] observed the same behavior by feeding Churra ewes with diets containing linseed oil and supplemented with increasing GP concentrations, 5 and 10 g/100 g of TMR respectively. Evaluations performed on milk did not evidence GP effects on the relative percentages of SFA, MUFA and PUFA. The presence of linseed oil determined for α-linolenic acid (C18:3 *cis*-9, *cis*-12, *cis*-15) a value close to 1% of total fatty acids, without variations in milk samples obtained from animals fed the GP supplementations. GP was instead effective in increasing the linoleic acid concentration, while no modifications have been registered for vaccenic acid, contrary to the previously mentioned studies. Similarly, Correddu et al. [103] evaluated the fatty acids profile of milk obtained from Sarda dairy ewes fed a dietary supplementation of grape seeds, alone or in combination with linseed. The authors reported an increase in concentration of oleic acid (C18:0) and linoleic acid in milk samples obtained from ewes which received only grape seed as dietary supplement; furthermore, in the same samples was shown a tendency to accumulate vaccenic acid and *cis*-9, *trans*-11 CLA in comparison with the control group. The increased concentration of linoleic acid was correlated with a presumable reduction in odd- and branched-chain fatty acids. The intake of polyphenols deriving from grape seeds may have contributed to this reduction, because of the supposed effect of these compounds on growth and activity of rumen microbiota [104]. The combined supplementation of grape seed and linseed was furthermore effective in improving the indices of health functionality in milk. In fact, both atherogenic and thrombogenic indices lowered, as evidence of a decreased potential risk of the onset of cardiovascular diseases in consumers following the intake of this food product.

### 4.3. Oxidative Stability of Ripened Dairy Products

The tendency of PUFA to undergo oxidation it is a topic of significant importance for the food industry, due to the fact that foods containing high concentrations of these compounds can undergo deterioration with detrimental effects on nutritional quality as well as a cause of concern for food safety [105]. The oxidative process has been suggested to start mainly as a result of the action of reactive species able to directly interact with C=C double bonds, with consequent release of peroxides [106].

As reported in the previous paragraph, the dietary intake of GP by lactating ruminants makes it possible to obtain milk and cheeses naturally enriched with PUFA, especially linoleic acid. Beyond the undoubted health benefits deriving from this finding, these food products should, however, be exposed to a greater predisposition to oxidation.

Ianni et al. [93] investigated this aspect by monitoring the extent of the oxidative damage in fresh and ripened cheeses through the evaluation thiobarbituric acid-reactive substances (TBARS), a method that uses the malondialdehyde (MDA) as marker of oxidative damage [107,108,109]. After 3 days from the cheese-making, the cheese obtained from cows fed the GP supplementation showed similar MDA values in comparison with cheese samples deriving from the control group. At the end of ripening (30 days) a very different picture was found, in which the cheese from the control group went through oxidation, whereas in the experimental cheese were defined MDA values similar with respect to those observed at the beginning of the ripening, despite the presence of greater concentrations of PUFA. This finding was attributed by authors to the presumable antioxidant action of phenolic compounds deriving from the GP supplemented to cows’ diet. In this regard, Santos et al. [110] reported a significant improvement of the reducing potential of milk obtained from lactating cows fed a dietary supplementation with ensiled GP. In addition to this, Correddu et al. [101] evidenced a significant reduction of the ratio between hydroperoxides and PUFA in milk collected from ewes that received a diet enriched with grape seeds.

There is no extensive literature on the evaluation of the oxidative state of dairy products obtained by feeding lactating ruminants with GP. However, in order to support the just-mentioned studies, it might be useful to focus attention also on the improvements in the oxidative stability of those cheeses produced by supplementing animals diet with matrices of vegetable origin, rich in bioactive compounds, especially polyphenols, credited of antioxidant properties [111].

### 4.4. Volatile Flavor Compounds and Sensorial Evaluations

The biochemical mechanisms that characterize cheese ripening have been widely treated and largely characterized. Briefly, such mechanisms can be divided into primary and secondary events. Primary events involve the metabolism of residual lactose, lactate and citrate, lipolysis and proteolysis, while secondary events are based on the metabolism of fatty acids and amino acids and directly contribute to the release of many volatile compounds (VOC), credited of high capacity to influence the cheese flavor [112].

It has been widely observed that the diet administered to lactating ruminants is commonly responsible for changes in the volatile profile of dairy products, both fresh and ripened [113,114,115,116]. It is, therefore, conceivable that compounds present in the diet, or secondary metabolites of the same, can be absorbed by the animal following digestion and then reach the mammary gland and be released into the milk. Following cheese manufacturing, some of these compounds could influence the biochemical mechanisms described above, both directly interacting with the enzymatic forms responsible for these events, or indirectly through the regulation of bacterial gene expression.

In this case, the literature includes sparse references regarding the effect of dietary GP intake on the VOC production in dairy products. In our knowledge, the only study of this type is the one conducted by Ianni et al. [100], in which lactating Friesian cows were fed for 60 days with a dietary supplementation of 10% of dried GP. The analysis of the VOC profile has been performed in fresh cheese (samples collected after 24 h form the cheese-making) and after 28 days of ripening. The most represented class of identified compounds was that of carboxylic acids, indicating the prevalence of the lipolytic process compared to the proteolytic one. The experimental feeding strategy was effective in reducing the concentrations of butanoic and hexanoic acids both in fresh and in ripened cheese samples. These compounds have been reported to significantly affect flavor formation in cheese, being associated with strong notes defined as sweaty, cheesy and rancid. This result was discussed by assuming a reduction of triglycerides degradation by microbial and endogenous milk enzymes, resulting in a limited production of free fatty acids (FFA) [117]. The second class of VOCs in order of abundance is represented by ethyl esters, which are associated with pleasant fruity, and floral notes with a low odor threshold. For that reason, their increase in concentration during ripening (from 4% at day 1 to 28% at the end of ripening) in cheese samples obtained as a consequence of the dietary GP supplementation is very interesting. In this study, a sensory analysis was also conducted. In fresh cheese, the dietary GP intake resulted effective in inducing a slightly darker coloring, a harder consistency, and a less sweet taste. No significant variations in terms of appearance, consistency, and taste were instead evidenced in ripened cheese samples.

## 5. Conclusions

As a result of what has been reported, the use of plant matrices as dietary supplements for lactating dairy ruminants seems to be desirable. Specifically, the development of feeding strategies based on the use of grape by-products has proven to be effective in the production of milk and dairy products characterized by implemented nutritional properties and improved oxidative stability, with several health benefits for consumers due to the presence of compounds credited of high biological value. In addition to this, the use of GP for enriching the livestock diet represents a viable way of recovering and valorizing the main by-product of the oenological industry, with undoubted environmental advantages.

## Figures and Tables

**Table 1:** Biological activities performed by phenolic compounds found in grape and its derivates.

Source/Compounds	Biological Function	References
Procyanidins	Antioxidant activity	[34]
	Free radical scavenging	[35]
	Anti-inflammatory activity	[37]
	Anti-carcinogenic activity	[38]
	Vasorelaxant activity	[36]
Proanthocyanidins	Anti-carcinogenic activity	[42]
Resveratrol	Anti-inflammatory activity	[43]
	Anti-carcinogenic activity	[43,44]
	Cardiovascular protection	[43]
	Fungicidal activity	[32]
	Regulation of lipid metabolism	[45]
Quercetin	Antioxidant activity	[48,49]
	Anti-inflammatory activity	[48,49,50]
	Anti-carcinogenic activity	[48,49,50]
	Regulation of lipid metabolism	[45]
Catechin	Free radical scavenging	[51]
	Antioxidant activity	[51]
	Anti-inflammatory activity	[53]
	Anti-carcinogenic activity	[54]
	Antibacterial	[52]
	Regulation of lipid metabolism	[45]
Gallic acid	Antioxidant activity	[48]
	Anti-inflammatory activity	[48]
	Anti-carcinogenic activity	[48]
Phenolic compounds (grape seed extract)	Inhibition of MMP-1 and MMP-9	[40]
	Inhibition of MMP-2 and MMP-2	[41]

MMP, matrix metallo-proteinase.
